# MiRNA-200a expression is inverse correlation with hepatocyte growth factor expression in stromal fibroblasts and its high expression predicts a good prognosis in patients with non-small cell lung cancer

**DOI:** 10.18632/oncotarget.10302

**Published:** 2016-06-27

**Authors:** Yongbing Chen, Menghua Du, Jin Wang, Pengfei Xing, Yongsheng Zhang, Feng Li, Xueguan Lu

**Affiliations:** ^1^ Department of Thoracic Surgery, the Second Affiliated Hospital of Soochow University, Suzhou, China; ^2^ Department of Oncology & Radiotherapy, the Second Affiliated Hospital of Soochow University, Suzhou, China; ^3^ Department of Pathology, the Second Affiliated Hospital of Soochow University, Suzhou, China; ^4^ Department of Radiation Oncology, Fudan University Shanghai Cancer Center, Shanghai, China

**Keywords:** lung cancer, stromal fibroblasts, miRNA-200a, hepatocyte growth factor, prognosis

## Abstract

Cancer-associated fibroblasts (CAFs) play an important role in favoring tumor progression. However, little is known concerning expression of miRNA-200a and its potential target gene hepatocyte growth factor (HGF) in CAFs. In the present study, we investigated expression levels and prognostic significance of miRNA-200a and HGF in stromal fibroblasts of non-small cell lung cancer (NSCLC), and evaluated the correlation between miRNA-200a and HGF. *In situ* hybridization and immunohistochemical staining were used to investigate expression levels of miRNA-200a and HGF in 134 formalin-fixed paraffin-embedded tumor specimens from clinical stage I -IIIA NSCLC, respectively. The results showed a significant inverse correlation existed between miRNA-200a and HGF expression level in stromal fibroblasts (χ^2^ = 21.778, *p* = 0.000). *In vitro*, the upregulation of miRNA-200a reduced expression of HGF protein in human CAFs. The 3-year overall survival (OS) rates with low and high miRNA-200a expression in stromal fibroblasts were 39.0% and 53.4%, respectively (χ^2^=4.25, *p*=0.039). The 3-year OS rates with low and high HGF expression in stromal fibroblasts were 60.3% and 31.8%, respectively (χ^2^=12.55, *p*=0.000). The multivariate analysis showed that clinical stage and HGF expression level in stromal fibroblasts were the independent predictive factors of OS. These results suggested that miRNA-200a expression was inverse correlation with HGF expression in stromal fibroblasts. High miRNA-200a and low HGF expression in stromal fibroblasts may predict a good prognosis in patients with NSCLC.

## INTRODUCTION

Lung cancer is the most common cause of cancer-related death for man and women, with an overall 5-year survival rate of 15%. Approximately 85% of lung cancer patients are NSCLC [1–2]. Tumor recurrence and distant metastases are the most common events appeared after surgical resection, which lead to poor prognosis [3]. In the past decades, treatment and molecular characteristics relating to prognosis of NSCLC were mainly focused on the cancer cells. However, recent studies revealed that the progression of tumors towards a malignant phenotype does not only depend on the cell-autonomous properties of cancer cells themselves but is also deeply influenced by tumor stroma [4]. Tumor stroma promotes tumor growth, invasion, metastases, and resistance to treatment as well as to mediate immune reaction against tumor cells [5]. The tumor stroma is formed by CAFs, macrophages, and other inflammatory cells as well as blood/lymphatic capillaries [6], and the CAFs is thought to be the main players among the cohabitating stromal cell types [7]. They favor tumor progression through secretion of soluble factors, as growth factors or inflammatory chemokines, as well as remodeling tumor extra-cellular matrix (ECM) and tumor metabolism, increasing of motility and stemness of cancer cells, and preparation of metastatic niche [4, 8]. HGF is a protein produced by CAFs that involves in promoting growth, motility and morphogenesis in cancer [9]. Grugan et al [10] found that HGF secreted by CAFs fosters the ability of transformed esophageal epithelial cells to invade into ECM. Until now, there had many studies to investigate the prognostic value of HGF expression in cancer cells for multiple cancer types [11–14]. However, little is known concerning the prognostic implication of HGF expression in stromal fibroblasts.

MicroRNAs (miRNAs) are small non-coding RNA gene products about 21-25 nucleotides long, which play a pivotal role as oncogenes or tumor suppressors in various cancers [15]. In the past few years, miRNAs have emerged as promising molecular factors with potential for clinical applications in cancer diagnosis and therapy [16]. MiRNA-200 is a family of tumor suppressor miRNAs consisting of five members (miRNA-200a, miRNA-200b, miRNA-200c, miRNA-429, and miRNA-141), which is significantly involved in inhibition of epithelial-to-mesenchymal transition (EMT), repression of cancer stem cells self-renewal and differentiation, modulation of cell division and apoptosis, and reversal of chemoresistance [16]. In previous studies, miRNA-200a expression has been shown to downregulate several prognostic markers for cancer patients, such as ZEB1, ZEB2, ATRX, DLC1, HFE and HNRNPA3 [17–18]. We used TargetScan analysis (http://www.targetscan.org) to predict that HGF may be one of the target gene regulated by miRNA-200a. However, not much is known regarding miRNA-200a expression in tumor stromal fibroblasts and the relationship between miRNA-200a and HGF in cancer. In present study, therefore, we separately investigate the expression levels of miRNA-200a and its potential target gene HGF in stromal fibroblasts and cancer cells from specimens of NSCLC, and evaluate the prognostic significance of these markers in patients with clinical stage I -IIIA NSCLC after curative resection.

## RESULTS

### Expression of miRNA-200a and HGF in stromal fibroblasts and cancer cells

**Figure 1:**
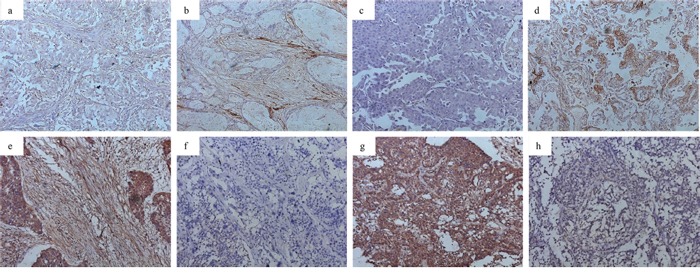
Expression of miRNA-200a and HGF in NSCLC (magnification ×200) **a.** low miRNA-200a in stromal fibroblasts; **b.** high miRNA-200a in stromal fibroblasts; **c.** low miRNA-200a in cancer cells; **d.** high miRNA-200a in cancer cells; **e.** high HGF expression in stromal fibroblasts; **f.** low HGF expression in stromal fibroblasts; **g.** high HGF expression in cancer cells; **h.** low HGF expression in cancer cells.

The expression of miRNA-200a and HGF was observed mainly in the cytoplasm. The percentages of high miRNA-200a expression in stromal fibroblasts and cancer cells were 59.7% (80/134) and 52.2% (70/134), respectively (Figure 1a–1d). The percentages of high HGF expression in stromal fibroblasts and cancer cells were 46.3% (62/134) and 82.1% (110/134), respectively (Figure 1e–1h). The analysis revealed a significant inverse correlation between miRNA-200a and HGF expression levels in stromal fibroblasts (χ^2^ = 21.778, *p* = 0.000; Table 1). No significant association was found between miRNA-200a and HGF expression levels in cancer cells (χ^2^ = 2.239, *p* = 0.135).

**Table 1:** Association between miRNA-200a and HGF expression levels in stromal fibroblasts

HGF expression level	No. of patients	miRNA-200a expression level		*p*
		Low (%)	High (%)	
Low	72	16 (22.2)	56 (77.8)	0.000
High	62	38 (61.3)	24 (38.7)	

**Figure 2:**
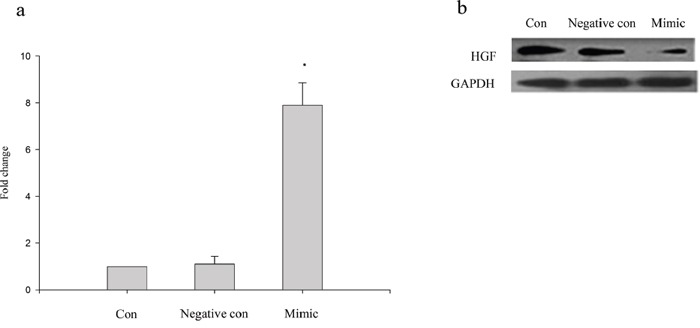
Expression of target gene HGF of miRNA-200a **a.** Real-time PCR analysis of miRNA-200a expression in human CAFs. Human CAFs transfected with miRNA-200a mimics and negative controls. The results indicated that miRNA-200a expression in higher in cells treated with miRNA-200a mimics; **b.** Western blotting analysis of CAFs treated with miRNA-200a mimics. Upregulation of miRNA-200a reduced expression of HGF.

To determine the regulatory role of miRNA-200a on HGF, we tested the effect of miRNA-200a elevation on HGF protein expression in human CAFs *in vitro*. Twenty-four hours after transfection, real-time PCR results showed that relative expression of miRNA-200a in the mimic-transfected group to be upregulated ~8-fold compared to control groups (p< 0.05; Figure 2a). The upregulation of miRNA-200a reduced the expression level of HGF protein in human CAFs. (Figure 2b)

### Association of miRNA-200a and HGF expression and clinicopathologic characteristics

With regard to age, gender, clinical stage, pathologic type, pathologic differentiation and vascular invasion, the significant difference that correlated with miRNA-200a expression in stromal fibroblasts was observed for gender (*p*=0.001) and pathologic type (*p*=0.004), respectively. The significant associations were found between miRNA-200a expression in cancer cells and age (*p*=0.027) and pathologic type (*p*=0.000) (Table 2). The significant difference that correlated with HGF expression in stromal fibroblasts was observed for gender (*p*=0.019) and clinical stage (*p*=0.005), respectively. The significant associations were found between HGF expression in cancer cells and pathologic differentiation (*p*=0.001) (Table 3).

**Table 2:** Association between miRNA-200a expression and clinicopathologic features

Variables	No. of patients	miRNA-200a expression in cancer cells			miRNA-200a expression in stromal fibroblasts		
		Low (%)	High (%)	*p*	Low (%)	High (%)	*P*
Gender							
Male	90	46 (51.1)	44 (48.9)	0.270	45 (50.0)	45 (50.0)	0.001
Female	44	18 (40.9)	26 (59.1)		9 (20.5)	35 (79.5)	
Age (years)							
≤63	72	28 (38.9)	44 (61.1)	0.027	28 (38.9)	44 (61.1)	0.722
>63	62	36 (58.1)	26 (41.9)		26 (41.9)	36 (58.1)	
Pathologic type							
Squamous carcinoma	56	38 (67.9)	18 (32.1)	0.000	30 (53.6)	26 (46.4)	0.004
Adenocarcinoma	65	23 (35.4)	42 (64.6)		22 (33.8)	43 (66.2)	
Adenosquamous carcinoma	7	1 (14.3)	6 (85.7)		2 (28.6)	5 (71.4)	
Large cell carcinoma	6	2 (33.3)	4 (66.7)		0 (0)	6 (100.0)	
Pathologic differentiation							
High	10	8 (80.0)	2 (20.0)	0.589	3 (30.0)	7 (70.0)	0.854
Median	91	36 (39.6)	55 (60.4)		38 (41.8)	53 (58.2)	
Low	33	20 (60.6)	13 (39.4)		13 (39.4)	20 (60.6)	
Clinical Stage*							
I	34	15 (44.1)	19 (55.9)	0.182	9 (26.5)	25 (73.5)	0.077
II	26	9 (34.6)	17 (65.4)		11 (42.3)	15 (57.7)	
IIIA	74	40 (54.1)	34 (45.9)		34 (45.9)	40 (54.1)	
Vascular invasion							
Negative	120	60 (50.0)	60 (50.0)	0.131	50(41.7)	70 (58.3)	0.348
Positive	14	4 (28.6)	10 (71.4)		4 (28.6)	10 (71.4)	

**Table 3:** Association between HGF expression and clinicopathologic features

Variables	No. of patients	HGF expression in cancer cells			HGF expression in stromal fibroblasts		
		Low (%)	High (%)	*p*	Low (%)	High (%)	*P*
Gender							
Male	90	19 (21.1)	71 (78.9)	0.169	42 (46.7)	48 (53.3)	0.019
Female	44	5 (11.4)	39 (88.6)		30 (68.2)	14 (31.8)	
Age (years)							
≤63	72	10 (13.9)	62 (86.1)	0.194	37 (51.4)	35 (48.6)	0.561
>63	62	14 (22.6)	48 (77.4)		35 (56.5)	27 (43.5)	
Pathologic type							
Squamous carcinoma	56	15 (26.8)	41 (73.2)	0.128	28 (50.0)	28 (50.0)	0.827
Adenocarcinoma	65	5 (7.7)	60 (92.3)		39 (60.0)	26 (40.0)	
Adenosquamous carcinoma	7	2 (28.6)	5 (71.4)		2 (28.6)	5 (71.4)	
Large cell carcinoma	6	2 (33.3)	4 (66.7)		3 (50.0)	3 (50.0)	
Pathologic differentiation							
High	10	0 (0)	10 (100.0)	0.001	4 (40.0)	6 (60.0)	0.897
Median	91	12 (13.2)	79 (86.8)		51 (56.0)	40 (44.0)	
Low	33	12 (36.4)	21 (63.6)		17 (51.5)	16 (48.5)	
Clinical Stage*							
I	34	5 (14.7)	29 (85.3)	0.898	24 (70.6)	10 (29.4)	0.005
II	26	6 (23.1)	20 (76.9)		16 (61.5)	10 (38.5)	
IIIA	74	13 (17.6)	61 (82.4)		32 (43.2)	42 (56.8)	
Vascular invasion							
Negative	120	21 (17.5)	99 (82.5)	0.719	66 (55.0)	54 (45.0)	0.392
Positive	14	3 (21.4)	11 (78.6)		6 (42.9)	8 (57.1)	

### Prognosis analysis

**Figure 3:**
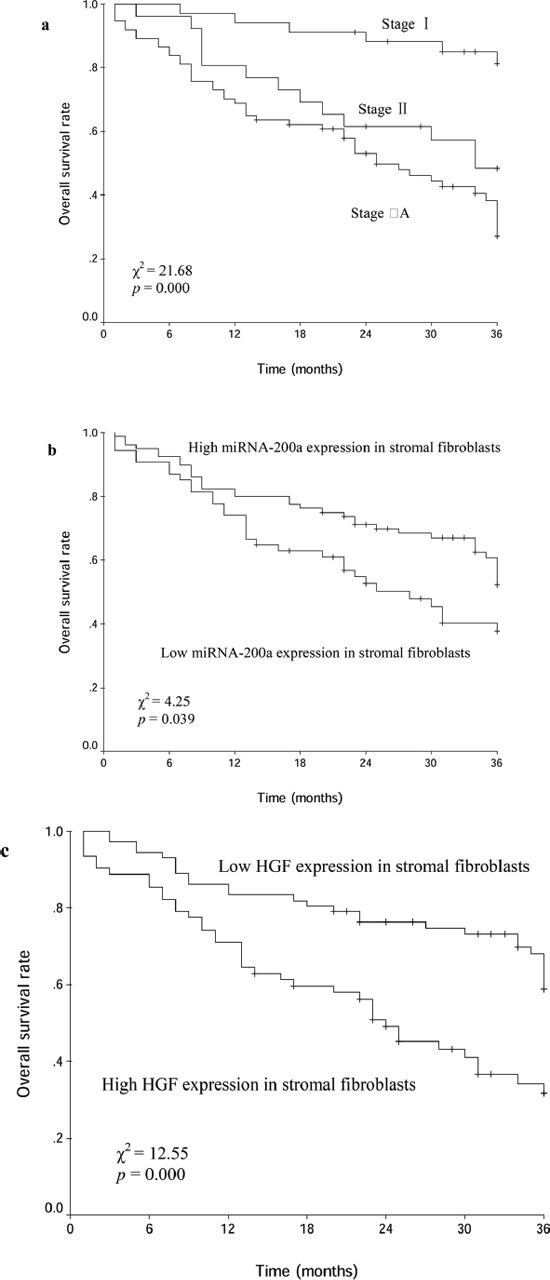
Kaplan-Meier curves for patients with clinical stage I- IIIA NSCLC after curative resection **a.** different clinical stage groups; **b.** miRNA-200a expression groups; **c.** HGF expression groups.

The median duration of follow-up for these patients was 28 months (range, 1-58 months). The Kaplan-Meier plots showed that the 3-year OS rate of all patients was 46.7%. The 3-year OS rates with clinical stage I, II and IIIA were 81.3%, 48.4% and 27.0%, respectively (χ^2^=21.68, *p*=0.000; Figure 3a). The 3-year OS rates with low and high miRNA-200a expression in stromal fibroblasts were 37.6% and 52.3%, respectively (χ^2^=4.25, *p*=0.039; Figure 3b). The 3-year OS rates with low and high HGF expression in stromal fibroblasts were 58.8% and 31.8% (χ^2^=12.55, *p*=0.000; Figure 3c). The multivariate analysis using by Cox proportional hazard model was fitted to investigate significant covariates including variables relation to OS: clinical stage, miRNA-200a and HGF expression in stromal fibroblasts. The clinical stage and HGF expression level in stromal fibroblasts were identified as the independent predictive factors of OS (Table 4). For 56 patients with squamous carcinoma, the 3-year OS rates with low and high miRNA-200a expression in stromal fibroblasts were 39.8% and 59.7%, respectively (χ^2^=1.86, *p*=0.172). The 3-year OS rates with low and high HGF expression in stromal fibroblasts were 67.4% and 30.6% (χ^2^=8.67, *p*=0.003). For 78 patients with non-squamous carcinoma, the 3-year OS rates with low and high miRNA-200a expression in stromal fibroblasts were 34.3% and 48.7%, respectively (χ^2^=2.68, *p*=0.102). The 3-year OS rates with low and high HGF expression in stromal fibroblasts were 54.0% and 32.1% (χ^2^=4.57, *p*=0.032). For 74 patients with the stage IIIA, the 3-year OS rates with low and high HGF expression in stromal fibroblasts were 43.6% and 13.0%, respectively (χ^2^=10.99, *p*=0.001). However, no significant association was found between low and high miRNA-200a/HGF expression levels in cancer cells with respect to OS in these patients, respectively (40.8% vs. 51.8%, χ^2^=2.30, *p*=0.130; 56.8% vs. 45.1%, χ^2^=0.04, *p*=0.843).

**Table 4:** Prognostic factors evaluated by univariate and multivariate analysis using Cox proportional hazard model (n=134)

Variables	Univariate analysis		Multivariate analysis	
	HR (95% CI*)	*p*	HR (95% CI*)	*P*
Sex (male/female)	0.646 (0.375-1.111)	0.114		
Age (>63/≤63)	0.979 (0.603-1.589)	0.930		
AJCC stage (I/II/IIIA)	2.191 (1.534-3.130)	0.000	2.091 (1.455-3.005)	0.000
Pathologic type (SCC/AC/ASC/LCC^#^)	1.152 (0.927-1.431)	0.201		
Pathologic differentiation (high/median/low)	0.905 (0.569-1.439)	0.674		
Vascular invasion (ngative/positive)	1.111 (0.507-2.433)	0.792		
miRNA-200a expression in cancer cells (low/high)	0.692 (0.426-1.124)	0.137		
miRNA-200a expression in stromal fibroblasts (low/high)	0.607 (0.373-0.986)	0.044	0.789 (0.473-1.315)	0.363
HGF expression in cancer cells (low/high)	1.070 (0.545-2.097)	0.845		
HGF expression in stromal fibroblasts (low/high)	2.364 (1.440-3.882)	0.001	1.892 (1.120-3.195)	0.017

# SCC/AC/ASC/LCC: squamous carcinoma/adenocarcinoma/adenosquamous carcinoma/large cell carcinoma

* CI: confidence interval

## DISCUSSION

The previous studies have revealed the dysregulation of miRNA-200a in various cancers, including bladder cancer, breast cancer, colorectal cancer, endometrial cancer, gastric cancer, prostate cancer, and lung cancer [17, 19–22]. However, the function of miRNA-200a may differ depending on cancer types. van Kempen et al [19] showed that decreased expression level of miRNA-200a was correlation with increasing thickness in primary melanomas and metastases. Moreover, progressive loss of miRNA-200a expression was associated with disease progression. Barron et al [20] also found that miRNA-200a overexpression can reduce prostate cancer cell growth and its lower expression may predict biochemical relapse following radical prostatectomy in prostate cancer patients. On the contrary, miRNA-200a was found to be high expression in ovarian cancer cells compared to normal human ovarian surface epithelial cells. Its high expression correlated with decreased progression-free survival [21–22]. Our results showed that miRNA-200a expression in cancer cells was correlated with pathologic type (*p*=0.000; Table 2). However, no significant association was found to be miRNA-200a expression level in cancer cells with respect to OS in patients with NSCLC. There had strong evidence in basic research to demonstrate that miRNA-200a inhibits EMT and suppresses lung cancer cell migration and invasion [16, 23]. Conversely, one function overexpression study has yielded conflicting results on the role of miRNA-200a in lung cancer cell migration [24]. These results indicate that the role of miRNA-200a in NSCLC is rather complex.

The present study tried to explore miRNA-200a expression level in stromal fibroblasts and evaluate its prognostic value in NSCLC. Our results showed that miRNA-200a expression of stromal fibroblasts in squamous carcinoma was higher than in non-squamous carcinoma (*p*=0.004; Table 2), as well as its expression in cancer cells. Furthermore, the 3-year OS rate was higher in patients with high miRNA-200a expression in stromal fibroblasts compared to those with low miRNA-200a expression (*p*=0.039; Figure 2b). However, no significant difference of OS was found between low and high miRNA-200a expression of stromal fibroblasts in patients with squamous carcinoma or non-squamous carcinoma, respectively (*p*=0.172; *p*=0.102). We think that the limited number of patients is the main cause of these results.

TargetScan analysis suggests that HGF may be one of the target gene regulated by miRNA-200a. Our results showed that a significant inverse correlation existed between miRNA-200a and HGF expression levels of stromal fibroblasts (*p* = 0.000; Table 1) in NSCLC specimen, whereas no significant association was found between miRNA-200a and HGF expression level in cancer cells (*p* = 0.135). Furthermore, *in vitro*, we confirmed overexpression of miRNA-200a decreased HGF protein level in human CAFs.

HGF, a ligand of c-Met proto-oncogene, has been reported to increase tumorigenetic, vascularizing, and motogenic effects on lung cancer. Most of previous studies have reported that HGF was commonly overexpressed in NSCLC and its overexpression was associated with poor prognosis in patients with NSCLC [25–26]. Our results showed that the percentage of high HGF expression in cancer cells was 82.1%. HGF expression level in cancer cells was associated with pathologic differentiation (*p*=0.001; Table 3). However, HGF expression level in cancer cells with respect to OS was no significant association. Similarly, Pan et al [14] also found that the relapse-free survival and OS had no significant differences between lung adenocarcinoma patients with high HGF expression and those with low HGF expression. In addition, we found that high HGF expression level in stromal fibroblasts of NSCLC was 46.3%, and its expression level was correlated with clinical stage (*p*=0.005). Furthermore, the 3-year OS rate was higher in patients with low HGF expression in stromal fibroblasts compared to those with high HGF expression in stromal fibroblasts. The multivariate analysis showed that HGF expression level in stromal fibroblasts was an independent prognostic factor for NSCLC patients. The OS rate was also higher in low HGF expression compared to high HGF expression of stromal fibroblasts in patients with squamous carcinoma and non-squamous carcinoma, respectively (*p*=0.003; *p*=0.032).

In conclusion, the results suggested that miRNA-200a expression was inverse correlation with HGF expression in stromal fibroblasts. High miRNA-200a and low HGF expression in stromal fibroblasts may predict a good prognosis in patients with NSCLC.

## MATERIALS AND METHODS

### Patients and specimen selection

The paraffin-embedded postoperative tissue specimens were obtained from the archives of department of pathology, the Second Affiliated Hospital of Soochow University, between January of 2010 and June of 2012. One hundred and thirty-four specimens of clinical stage I-IIIA NSCLC were retrieved. Approval for current project was obtained from the local ethics committee together with written informed consent from each patient.

The main characteristics of 134 patients were summarized in Table 5. Ages of all patients in this study range from 31 to 81 years (median, 63 years). According to AJCC/UICC (6^th^ edition), there enrolled 34 patients with stage I, 26 patients with stage II, and 74 patients with stage IIIA. All patients underwent curative resection, of which 21 patients received segmentectomy, 98 patients received lobectomy and 15 patients received pneumonectomy. Systemic adjuvant treatment was administered to 112 patients. The chemotherapeutic regimen was cisplatin-based doublet. Twenty-five patients received thoracic postoperative radiotherapy.

**Table 5:** Patient characteristics

Characteristic	No. of patients (%)
Patients	134 (100.0)
Median age = 63 years (range 31-81)	
Gender	
Male	90 (67.2)
Female	44 (32.8)
Pathologic type	
Squamous carcinoma	56 (41.8)
Adenocarcinoma	65 (48.5)
Adenosquamous carcinoma	7 (5.2)
Large cell carcinoma	6 (4.5)
Pathologic differentiation	
High	10 (7.5)
Median	91 (67.9)
Low	33 (24.6)
Clinical Stage*	
I	34 (25.4)
II	26 (19.4)
IIIA	74 (55.2)
Vascular invasion	
Negative	120 (89.6)
Positive	14 (10.4)
Treatment modality-Curative resection	
Yes	134 (100.0)
No	0 (0)
Treatment modality-Adjuvant chemotherapy	
Yes	112 (83.6)
No	22 (16.4)
Treatment modality-Postoperative radiotherapy	
Yes	25 (18.7)
No	109 (81.3)

* According to UICC/AJCC (6^th^ edition) stage system

### *In situ* hybridization (ISH) staining

Three serial slides, each 3-5 um thick, were cut from paraffin-embedded tissue. One slide was used to give HE staining again. Immunohistochemical staining for HGF was performed on the second slide. The third slide was used to evaluate miRNA-200a expression by ISH. In brief, the slides were incubated at 60^°^C for 1 hour, deparaffinized in xylene, and rehydrated with graded alcohol washes. Slides were then washed three times with diethyl pyrocarbonate-treated PBS, digested with 5 ug/ml proteinase K at 37 ^°^C for 30 minutes, washed then dehydrated in graded alcohol. Slides were hybridized at 55^°^C for 2 hours with 50 nmol/L locked nucleic acid-modified digoxigenin-labeled probes for miRNA-200a (Boster, Wuhan, China). After stringency washes (5x, 1x, 0.2x SSC), slides were placed in blocking solution for 1 hour at room temperature followed by incubation in alkaline phosphatase conjugated anti-DIG Fab fragment solution at 37^°^C for 2 hours. Antibody signal was amplified with 4-nitro-blue tetrazolium and 5-bromo-4-chloro-3′-indolylphosphate substrate (Roche, Mannheim, Germany). Finally, hematoxylin was used as a light nuclear counterstain.

The intensity histoscore with the following categories according to Donahue's description was used: 0 negative, 1 weakly positive, 2 moderately positive, and 3 strongly positive [27]. The final score between 0-1 was determined as low expression, and score between 2-3 was determined as high expression.

### Immunohistochemical staining

Immunohistochemical staining was performed by two-step procedure. Upon rehydration as above, the slides were subjected to antigen retrieval by pressure-cooking for 15 minutes. Endogenous peroxidase activity was neutralized using peroxide block placement on the slides for 10 minutes at room temperature. The slides were then incubated with anti-HGF polyclonal antibody (ab83760, Abcam, Cambridge, MA; diluted 1: 200) at 4^°^C overnight. This was followed by incubation with peroxidase-conjugated polymer (ChemMate EnVision / HRP; Gene Tech, Shanghai, China) for 30 minutes at room temperature. The chromogen reaction was developed in 3,3′-diaminobenzidine (DAB; Gene Tech, Shanghai, China) tetrahydrochloride for 5 minutes. Finally, hematoxylin was used as a light nuclear counterstain.

The percentage of positive-staining cells were graded on a scale of 0-3, with less than 5% positive-staining cells as grade 0, 5-25% as grade 1, 26-50% as grade 2, and more than 50% as grade 3. The intensity of staining also graded on a scale of 0-2, with negative to weak intensity as grade 0, weak-moderate intensity as grade 1, and moderate to strong intensity as grade 2. Finally, the score of percentage and intensity was multiplied. The final score between 0-2 was determined as low expression, and score higher than 2 was determined as high expression.

### Cell culture and transfection

The fresh specimen from the resected lung adenocarcinoma tissues was used to culture CAFs. The process of primary culture and identification of CAFs has been made a detailed description in our previous study [28]. The CAFs were used to conduct experiment after 3-5 passages following primary culture.

The sequence of miRNA-200a mimics and negative control are listed as below:

5′-UAACACUGUCUGGUAACGAUGUAUCGUUACCAGACAGUGUUAUU-3′ and 5′-UUCUCCGAACGUGUCACGUTTACGUGACACGUUCGGAGAATT-3′

To transfect human CAFs, 100-pmol mimics of miRNA-200a (Ribobio, Guangzhou, China) and negative control in 200ul of serum-free medium were mixed with 5ul of Lipofectamine 2000 transfection reagent (Ribobio), dissolved in 200ul of the same medium, respectively. The resulting transfection solutions were then added to each well containing 1.6 ml of the medium. The culture was replaced with differentiation medium after 6. 0 h.

### Quantative real-time PCR analysis

Total RNA was harvested from human CAFs using the miRNeasy Mini kit (Qiagen GmbH, Hilden, Germany) according to the manufacturer's recommendations. Total RNA was subsequently reverse transcribed to cDNA with the stem-loop reverse transcription primer (Beijing Genomics Institute, Beijing, China) for miRNA-200a detection. The primer sequences are listed as below: (1) miRNA-200a RT: 5′-GTCGTATCCAGTGCGTGTCGTGGAGTCGGCAATTGCACTGGATACGACACATCGT-3′; (2) miRNA-200a qPCR-F: 5′-GGGGTAACACTGTCTGGTAG-3′. Real-time -PCR was carried out using SYBR Premix Ex Taq™ (Takara Biotechnology, Dalian, China). The reactions were placed in a 96-well plate using a preheated real-time instrument (ABI 7500HT; Applied Biosystems Life Technologies). The relative levels of expression were quantified and analyzed using Bio-Rad iCycler iQ software (Bio-Rad Laboratories, Hercules, CA, USA). Ct values were used to calculate the RNA expression levels. The amount of miRNA-200a expression (2^−ΔΔ^Ct) was normalized using the endogenous U6 reference.

### Western blotting analysis

Briefly, human CAFs were lysed in a buffer consisting of 50 mM Tris-HCl (pH 6.8). After brief sonication, lysates were clarified by centrifugation at 12 000 × g for 10 min at 4^°^C, and protein content in the supernatant was measured according to the Bradford method. Aliquots (80-100 ug of protein per lane) of total protein were separated by 8% SDS-polyacrylamide gel electrophoresis and blotted onto nitrocellulose transfer membranes (Beyotime, China). Each membrane was blocked with 5% non-fat dry milk in TBS-T for 1.5 h at room temperature, followed by incubation with the antibody HGF (diluted 1: 1000) overnight at 4^°^C. After extensive washing with TBST, each membrane was further incubated with horseradish peroxidase-conjugated anti-rabbit secondary antibodies (1:5000) for 2.0 h at room temperature in TBST containing 5% non-fat dry milk. Detection was performed using an enhanced chemiluminescence reagent (Beyotime, China), according to the manufacturer's protocol.

### Statistical analysis

The relationships between miRNA-200a and HGF expression in cancer cells or stromal fibroblasts and clincopathologic characteristics were examined by Spearmann correlation coefficient test. OS rates were performed by the Kaplan-Meier method and log-rank test. The Cox proportional hazard model was used for univariate and multivariate analysis. Overall survival duration was defined from the day of surgery to the day of death or last follow-up. For all tests, a two-sided p< 0.05 was considered significant.
